# Syntrophomonas wolfei Uses an NADH-Dependent, Ferredoxin-Independent [FeFe]-Hydrogenase To Reoxidize NADH

**DOI:** 10.1128/AEM.01335-17

**Published:** 2017-09-29

**Authors:** Nathaniel A. Losey, Florence Mus, John W. Peters, Huynh M. Le, Michael J. McInerney

**Affiliations:** a Department of Plant Biology and Microbiology, University of Oklahoma, Norman, Oklahoma, USA; b Department of Chemistry and Biochemistry, Institute of Biological Chemistry, Washington State University, Pullman, Washington, USA; Kyoto University

**Keywords:** Syntrophomonas, hydrogenase, methanogenesis, syntrophs

## Abstract

Syntrophomonas wolfei syntrophically oxidizes short-chain fatty acids (four to eight carbons in length) when grown in coculture with a hydrogen- and/or formate-using methanogen. The oxidation of 3-hydroxybutyryl-coenzyme A (CoA), formed during butyrate metabolism, results in the production of NADH. The enzyme systems involved in NADH reoxidation in S. wolfei are not well understood. The genome of S. wolfei contains a multimeric [FeFe]-hydrogenase that may be a mechanism for NADH reoxidation. The S. wolfei genes for the multimeric [FeFe]-hydrogenase (*hyd1ABC*; SWOL_RS05165, SWOL_RS05170, SWOL_RS05175) and [FeFe]-hydrogenase maturation proteins (SWOL_RS05180, SWOL_RS05190, SWOL_RS01625) were coexpressed in Escherichia coli, and the recombinant Hyd1ABC was purified and characterized. The purified recombinant Hyd1ABC was a heterotrimer with an αβγ configuration and a molecular mass of 115 kDa. Hyd1ABC contained 29.2 ± 1.49 mol of Fe and 0.7 mol of flavin mononucleotide (FMN) per mole enzyme. The purified, recombinant Hyd1ABC reduced NAD^+^ and oxidized NADH without the presence of ferredoxin. The HydB subunit of the S. wolfei multimeric [FeFe]-hydrogenase lacks two iron-sulfur centers that are present in known confurcating NADH- and ferredoxin-dependent [FeFe]-hydrogenases. Hyd1ABC is a NADH-dependent hydrogenase that produces hydrogen from NADH without the need of reduced ferredoxin, which differs from confurcating [FeFe]-hydrogenases. Hyd1ABC provides a mechanism by which S. wolfei can reoxidize NADH produced during syntrophic butyrate oxidation when low hydrogen partial pressures are maintained by a hydrogen-consuming microorganism.

**IMPORTANCE** Our work provides mechanistic understanding of the obligate metabolic coupling that occurs between hydrogen-producing fatty and aromatic acid-degrading microorganisms and their hydrogen-consuming partners in the process called syntrophy (feeding together). The multimeric [FeFe]-hydrogenase used NADH without the involvement of reduced ferredoxin. The multimeric [FeFe]-hydrogenase would produce hydrogen from NADH only when hydrogen concentrations were low. Hydrogen production from NADH by Syntrophomonas wolfei would likely cease before any detectable amount of cell growth occurred. Thus, continual hydrogen production requires the presence of a hydrogen-consuming partner to keep hydrogen concentrations low and explains, in part, the obligate requirement that S. wolfei has for a hydrogen-consuming partner organism during growth on butyrate. We have successfully expressed genes encoding a multimeric [FeFe]-hydrogenase in E. coli, demonstrating that such an approach can be advantageous to characterize complex redox proteins from difficult-to-culture microorganisms.

## INTRODUCTION

The model syntrophic bacterium Syntrophomonas wolfei oxidizes butyrate to acetate with the concomitant production of hydrogen and formate (1). During syntrophic butyrate metabolism, the hydrogen- and/or formate-using methanogen maintains low levels of hydrogen and formate so that butyrate oxidation remains thermodynamically favorable (2, 3). Butyrate oxidation by S. wolfei involves two different oxidation-reduction reactions, generating electrons at two different redox potentials, both of which are used to produce hydrogen or formate (4–6). The first set of electrons is generated during the oxidation of butyryl-coenzyme A (butyryl-CoA) to crotonyl-CoA. Due to the high redox potential of this electron pair (E^0′^ = −125 mV), it has been proposed that membrane complexes utilizing chemiosmotic energy are required for hydrogen or formate production (4, 5, 7–9). The second pair of electrons is generated from the oxidation of 3-hydroxybutyryl-CoA to acetoacetyl-CoA (E ^0′^ = −250 mV) and is used to reduce NAD^+^ to NADH (E^0′^ = −320 mV). NADH reoxidation must be coupled to hydrogen or formate production during syntrophic butyrate metabolism. Under the low hydrogen partial pressures maintained by hydrogenotrophic methanogens (<10 Pa), the redox potential (E′) of hydrogen is about −260 mV, making hydrogen production directly from NADH thermodynamically feasible (3, 10).

The analysis of the S. wolfei genome identified a multimeric [FeFe]-hydrogenase encoded by genes (*hyd1ABC*; SWOL_RS05165, SWOL_RS05170, SWOL_RS05175) that shared a sequence identity similar to those of confurcating [FeFe]-hydrogenases (4, 10, 11), a NADPH-linked [FeFe]-hydrogenase (12), and NADH-dependent formate dehydrogenases (13). Most examples of multimeric [FeFe]-hydrogenases have been reported to function in either electron confurcation or the reverse (bifurcating) reaction. Confurcating [FeFe]-hydrogenases couple the unfavorable production of hydrogen from NADH with the favorable production of hydrogen from reduced ferredoxin (11), utilizing equimolar amounts of NADH and reduced ferredoxin to produce hydrogen_,_ as was first demonstrated in the multimeric [FeFe]-hydrogenase from Thermotoga maritima (11). Additional multimeric [FeFe]-hydrogenases have been studied in Acetobacterium woodii (14), Moorella thermoacetica (15), and Ruminococcus albus (16).

The beta-oxidation of butyrate by S. wolfei does not directly result in the formation of reduced ferredoxin. Thus, the metabolism of S. wolfei differs from the metabolism of organisms with reported confurcating/bifurcating [FeFe]-hydrogenases, which have ferredoxin-dependent oxidoreductases, such as pyruvate:ferredoxin oxidoreductase, that produce reduced ferredoxin during substrate degradation (11, 14–16). One potential source of reduced ferredoxin in S. wolfei is a putative Fix system (nitrogen fixation), which could produce reduced ferredoxin from the oxidation of NADH (4). However, the use of Fix to produce reduced ferredoxin would place a high energy demand on an organism that uses growth reactions that operate close to thermodynamic equilibrium (17). In the absence of an identifiable source of reduced ferredoxin and with syntrophic growth conditions that would be permissive for hydrogen production from NADH, it is likely that Hyd1ABC functions as a nonconfurcating, NADH-dependent [FeFe]-hydrogenase.

We cloned and expressed the genes of a multimeric [FeFe]-hydrogenase (SWOL_RS05165, SWOL_RS05170, SWOL_RS05175) from S. wolfei and characterized the recombinant protein in order to understand the mechanisms by which S. wolfei reoxidizes NADH. To obtain an active enzyme, it was also necessary to coexpress the genes required for [FeFe]-hydrogenase maturation (SWOL_RS05180, SWOL_RS05190, SWOL_RS01625). This strategy has been used successfully in the past to produce active [FeFe]-hydrogenases using Escherichia coli (18, 19) and allowed the production of an enzyme with the activity of a NADH-dependent [FeFe]-hydrogenase (Hyd1ABC: SWOL_RS05165, SWOL_RS05170, SWOL_RS05175 gene products).

## RESULTS

### Purification and molecular weight.

**FIG 1:**
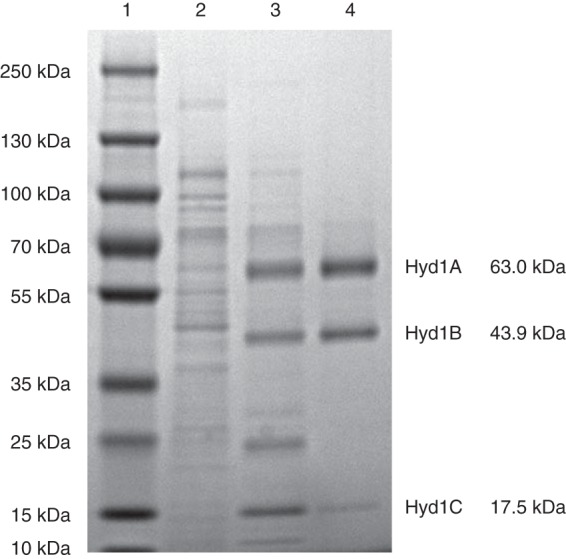
SDS-PAGE of the NADH-dependent [FeFe]-hydrogenase Hyd1ABC. Lanes: 1, molecular mass markers; 2, E. coli cell extract; 3, HisTrap HP; 4, UNO-Q1. The displayed molecular masses of Hyd1A-C peptides are predicted from the amino acid sequence.

**FIG 2:**
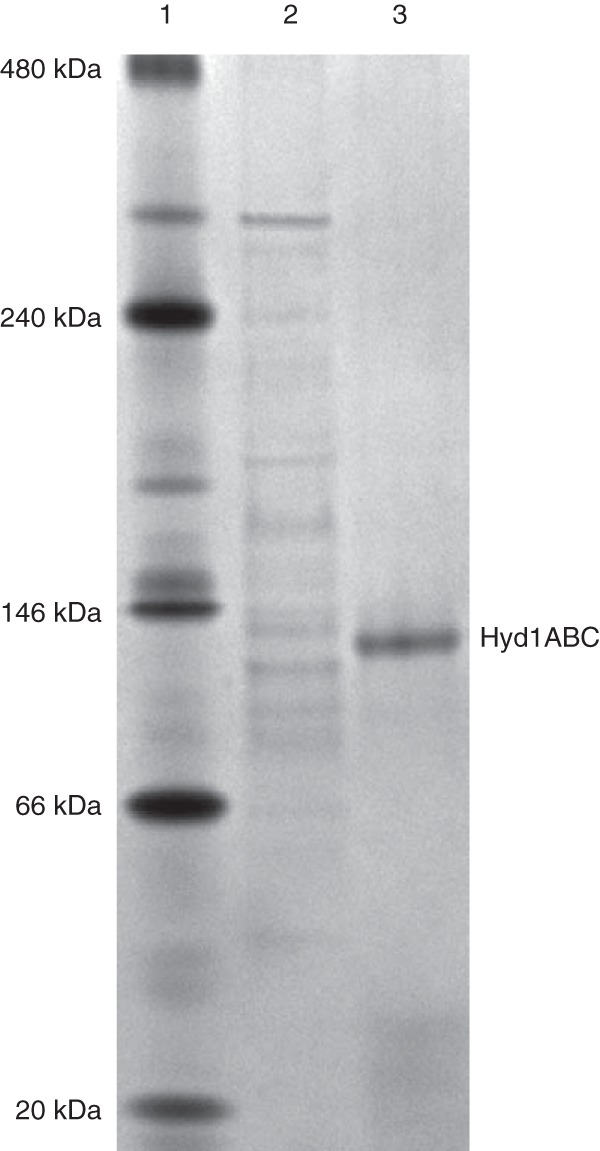
Native PAGE of the NADH-dependent [FeFe]-hydrogenase Hyd1ABC. Lanes: 1, native molecular mass markers; 2, E. coli cell extract; 3, UNO-Q1 fraction of the purified Hyd1ABC.

The recombinant gene products of SWOL_RS05165, SWOL_RS05170, and SWOL_RS05175 were purified to apparent homogeneity using nickel affinity and anion exchange chromatography, resulting in an apparent yield of 12.7% (Table 1). Approximately 0.55 mg of purified recombinant enzyme was obtained from 10.8 g (wet weight) of cells. Sodium dodecyl sulfate-polyacrylamide gel electrophoresis (SDS-PAGE) analysis showed that the purified recombinant enzyme consisted of three subunits with molecular weights that were consistent with molecular weights predicted from the sequence of encoding genes, namely, 63 kDa compared to the predicted 63.0 kDa (HydA1), 43 kDa compared to the predicted 43.9 kDa (HydB1), and 13 kDa compared to the predicted 17.5 kDa (HydC1) with the included molecular weight of the His tag (MGSSHHHHHHSQDP, 1.6 kDa) (Fig. 1). Native PAGE analysis showed that the purified enzyme migrated as a single band (Fig. 2), and size exclusion chromatography gave a molecular mass of 115 kDa. The single visible band from native gel electrophoresis when subjected to peptide analysis showed high coverage scores for the intended peptides (Table S1). Based on the subunit analysis and the native molecular weight, Hyd1ABC is a heterotrimer with an αβγ configuration and a molecular mass of 124.5 kDa as predicted by the sequence of the encoding genes. Iron content was determined to be 29.2 ± 1.49 mol of Fe per mole of enzyme, which matches well with the predicted 30 mol Fe per mole of enzyme from conserved domain analysis, which indicated five [4Fe-4S] clusters, two [2Fe-2S] clusters, and the six Fe in the [H] cluster. The flavin content was 0.7 mol of flavin mononucleotide (FMN) per mole of enzyme, which agrees with the single flavin-binding site in the HydB subunit, predicted from conserved domain analysis.

**TABLE 1:** Purification of the NADH-dependent [FeFe]-hydrogenase Hyd1ABC

Fraction	Protein (mg)	Activity H_2_ → MV_ox_ (U · mg^−1^)^*a*^		Purification (fold)	Yield (%)
		Specific	Total		
Cell extract	670	16.8	11,200	1	100
HisTrap HP	4.0	655	2,620	38.9	23.4
UNO-Q1	0.43	3,340	1,430	199	12.7

a Activity was determined at 37°C, pH 7.5; 1 unit of activity (U) equals 2 μmol of electrons transferred per minute. MV_ox_, oxidized methyl viologen.

### Activity.

**FIG 3:**
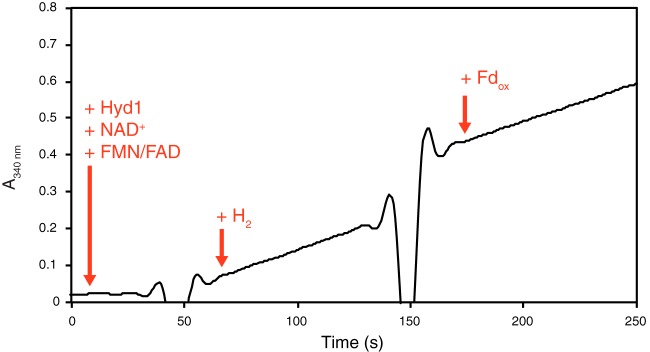
Reduction of NAD^+^ by the purified, NADH-dependent [FeFe]-hydrogenase Hyd1ABC with hydrogen as the electron donor. Reactions were performed in 1.5-ml, sealed, anaerobic cuvettes containing 0.5 ml of 50 mM potassium phosphate buffer (pH 7.5 at 37°C), 5 μM FAD, 5 μM FMN, 2 mM DTE, 1 mm NAD^+^, and 0.11 μg of Hyd1ABC. Clostridial ferredoxin (Fd_ox_) was added at a concentration of 10 μM. The change in absorbance (ΔA_340_) before the addition of hydrogen was 0.129 A · min^−1^, and after the addition of oxidized ferredoxin it was 0.125 A · min^−1^.

**FIG 4:**
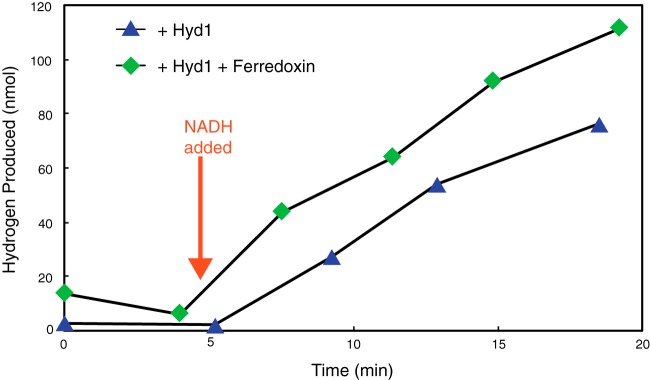
The effect of NADH and reduced ferredoxin on hydrogen production by Hyd1ABC. Reactions were performed in 6.5-ml serum bottles containing 0.5 ml of 100 mM potassium phosphate buffer (pH 7.5 at 37°C), 5 μM FAD, 5 μM FMN, 2 mM DTE, 1 mM NADH, and 1.3 μg of Hyd1ABC. A ferredoxin reduction system as indicated by “+ Ferredoxin” consisted of 10 mM pyruvate, 1 mM CoA, 0.1 mM thiamine pyrophosphate, 5 μM clostridial ferredoxin, and 0.1 U of clostridial pyruvate:ferredoxin oxidoreductase. NADH (1 mM) was added immediately after sampling time points indicated by the red arrow.

The purified recombinant Hyd1ABC had high hydrogen-dependent methyl viologen-reducing activity and reduced methyl viologen-oxidizing activity (Table 2), typical for many [FeFe]-hydrogenases. The purified recombinant Hyd1ABC also had high NAD^+^-reducing activity with hydrogen as the electron donor (specific activity of 94.5 U · mg^−1^ at 37°C) and catalyzed hydrogen production from NADH with a specific activity of 6.6 U · mg^−1^ at 37°C (Table 2). The enzyme neither oxidized NADPH nor reduced NADP^+^ under any condition tested. Neither the rate of NAD^+^ reduction nor the rate of NADH-dependent hydrogen production changed when oxidized or reduced clostridial ferredoxin, respectively, was added (Fig. 3 and 4). In addition, the purified recombinant Hyd1ABC did not reduce clostridial ferredoxin either alone or in the presence of NAD^+^ with hydrogen as the electron donor. Hyd1ABC did not produce hydrogen from reduced clostridial ferredoxin in the absence of NADH, and the amount of hydrogen produced when both NADH and reduced ferredoxin were present was similar to that produced when only NADH was present (Fig. 4). The above-described experiments were repeated with the purified recombinant S. wolfei ferredoxin encoded by SWOL_RS10890 with results similar to those using the clostridial ferredoxin. That is, the recombinant S. wolfei ferredoxin did not appear to affect the rate of NAD^+^ reduction or NADH oxidation. In addition, Hyd1ABC did not reduce the recombinant S. wolfei ferredoxin with hydrogen as the electron donor and did not produce hydrogen with reduced recombinant S. wolfei ferredoxin as the electron donor. The kinetics of NAD^+^ reduction with hydrogen, where the NAD^+^ concentration was varied, indicated a *K_m_* for NAD^+^ of 520 μM, a V_max_ of 196 U · mg^−1^, and a k_cat_ of 406.7 s^−1^, assuming a single catalytic site per a molecular mass of 124.5 kDa (see Fig. S1 in the supplemental material). Hyd1ABC specific activities for methyl viologen and NAD^+^ reduction and for NADH oxidation were similar to those reported for other multimeric [FeFe]-hydrogenases; however, the *K_m_* for reduction of NAD^+^ by Hyd1ABC was higher than that of other multimeric [FeFe]-hydrogenases (Table S2).

**TABLE 2:** Specific activities of the purified NADH-dependent [FeFe]-hydrogenase Hyd1ABC

Reaction^*a*^	Hydrogenase activity (U · mg^−1^)^*b*^
H_2_ → MV_ox_	571
H_2_ → NAD^+^	94.5
H_2_ → NAD^+^ + Fd_ox_	92.1^*c*^
H_2_ → NADP^+^	<0.01
H_2_ → NADP^+^ + Fd_ox_	<0.01
H_2_ → Fd_ox_	<0.01
MV_red_ → H_2_	24.3
NADH → H_2_	6.6
NADH + Fd_red_ → H_2_	6.0
NADPH → H_2_	<0.01
NADPH + Fd_red_ → H_2_	<0.01
Fd_red_ → H_2_	<0.01

a MV_ox_, oxidized methyl viologen; FD_ox_, oxidized ferredoxin; MV_red_, reduced methyl viologen; FD_red_, reduced ferredoxin.

b Activity was determined at 37°C, pH 7.5; 1 unit of activity (U) equals 2 μmol of electrons transferred per minute.

c Activity is the rate of NAD^+^ reduction at 340 nm in the presence of Fd_ox_ and not the rate of Fd_ox_ reduction (at 430 nm), which was not observed to occur.

In the above-described assays, the purified Hyd1ABC produced hydrogen from NADH under conditions where only a small amount of NADH would be oxidized to NAD^+^ during the time course of the assay and thus the ratio of NADH to NAD^+^ would remain high. To determine whether Hyd1ABC could produce hydrogen from NADH under more physiological conditions, we varied the ratio of NADH to NAD^+^ and the pH but kept the total pyridine nucleotide concentration constant at 5 mM (Table 3). In each case, more hydrogen was produced with a NADH/NAD^+^ ratio of 1.5 than with a ratio of 1.0. More hydrogen was also produced at pH 6.5 than at pH 7.0. Hydrogen partial pressures varied from 17.0 Pa, with a range of 11.3 Pa, to 40.0 Pa with a range of 6.9 Pa, with higher partial pressures observed at lower pH values and higher NADH/NAD^+^ ratios. Final hydrogen partial pressures detected in these experiments were below those predicted at thermodynamic equilibrium for the given NADH/NAD^+^ ratios. With the given liquid and gas volumes, a NADH/NAD^+^ ratio of 1.5, and a pH of 7.0, the redox potential (E′) of the NADH/NAD^+^ pair should be greater than −320 mV, even with the shift in NADH/NAD^+^ ratio as NADH is oxidized. The predicted equilibrium hydrogen partial pressure is in excess of 70 Pa for these conditions; however, the measured hydrogen partial pressure was lower, at 25.9 Pa, with a range of 0.8, suggesting that thermodynamic equilibrium was not reached.

**TABLE 3:** Equilibrium hydrogen partial pressures produced by the NADH-dependent [FeFe]-hydrogenase Hyd1ABC at different pH values and NADH/NAD^+^ ratios^*a*^

Condition	NADH/NAD^+^ ratio	Mean (range) hydrogen partial pressure (Pa)	Hydrogen produced (nmol)
pH 6.5, 2.0 mM NAD^+^, 3.0 mM NADH	1.5	40.0 (6.9)	95.4
pH 6.5, 2.5 mM NAD^+^, 2.5 mM NADH	1.0	21.8 (8.7)	51.8
pH 7.0, 2.0 mM NAD^+^, 3.0 mM NADH	1.5	25.9 (0.8)	61.9
pH 7.0, 2.5 mM NAD^+^, 2.5 mM NADH	1.0	17.0 (11.3)	40.4

a Assays were performed in 6.5-ml serum bottles with 0.5 ml of liquid volume containing 0.86 μg of protein and 6 ml of headspace.

### Hydrogen levels in growing coculture.

Hydrogen partial pressures measured during growth of S. wolfei in coculture with Methanospirillum hungatei on either butyrate or crotonate were between 4.6 and 18.0 Pa (Fig. S2), with the highest concentrations observed at day four and lower concentrations observed during the later stages of growth. Hydrogen partial pressures measured for pure culture S. wolfei grown on crotonate were between 27.4 and 40.0 Pa.

## DISCUSSION

The identification of a NADH-dependent [FeFe]-hydrogenase in S. wolfei provides an explanation for how electrons derived from 3-hydroxybutyryl-CoA oxidation are used to make hydrogen during syntrophic growth. Stoichiometrically, all electrons derived from the oxidation of butyrate are converted to either hydrogen or formate, as S. wolfei does not produce any other reduced end products during syntrophic butyrate metabolism (20, 21). The electrons generated by the oxidation of 3-hydroxybutyryl-CoA to acetoacetyl-CoA are used to reduce NAD^+^ to NADH (6). The presence of genes encoding a multimeric [FeFe]-hydrogenase and NADH-linked formate dehydrogenase in the genome of S. wolfei (4) suggested that NADH could be reoxidized using these enzyme systems. Hyd1 represents the only hydrogenase encoded within the genome of S. wolfei predicted to catalyze NADH oxidation. Of the two other hydrogenases present within the S. wolfei genome, Hyd2, encoded by SWOL_RS09950, is a membrane-bound [FeFe]-hydrogenase believed to interact with the menaquinone pool (4, 7, 8), while Hyd3, encoded by SWOL_RS12620, is a monomeric type [FeFe]-hydrogenase (4), which has not been reported to interact with NADH (22). The *hyd1A* gene was upregulated under coculture conditions on butyrate compared to coculture conditions on crotonate (9), and Hyd1ABC was confirmed to be present by proteomic techniques (5, 7) during syntrophic coculture growth on butyrate with M. hungatei. Thus, Hyd1ABC along with NADH-linked formate dehydrogenases are the likely routes of NADH reoxidation in S. wolfei.

The possibility that S. wolfei utilizes a NADH-dependent hydrogenase was suggested previously due to the closeness of the redox potentials of 3-hydroxybutyryl-CoA/acetoacetyl-CoA (E^0′^ = −250 mV), NADH/NAD^+^ (E^0′^ = −320 mV), and H_2_/H^+^ at 1 Pa H_2_ (E′ = −261 mV) (3). The amino acid sequence comparisons suggested that Hyd1ABC might be a confurcating hydrogenase (4, 10). Confurcating/bifurcating [FeFe]-hydrogenses produce hydrogen from NADH (E^0′^ = −320 mV) and reduced ferredoxin (E^0′^ = −398 mV or less) (11, 23). Coupling the unfavorable hydrogen production from NADH with the favorable hydrogen production from reduced ferredoxin would allow hydrogen production at partial pressures greater than 1,000 Pa at equilibrium (equivalent to E′ = −367 mV). The NADH/NAD^+^ midpoint potential (E^0′^ = −320 mV) would allow for a much lower hydrogen partial pressure of close to 70 Pa at equilibrium. Using fixed NADH/NAD^+^ ratios of 1.0 and 1.5, Hyd1ABC produced partial pressures of hydrogen of 17 to 40 Pa (Table 3). NADH/NAD^+^ ratios reported for organisms grown under anaerobic conditions can vary, with some values lower than those tested here, for example, 0.27 for Clostridium kluyveri, 0.29 for Clostridium welchii, and 0.4 for E. coli (24), while other studies have reported higher ratios of 0.75 for E. coli (25) and 1.16 for Enterococcus faecalis (26). Thus, the production of hydrogen by Hyd1ABC from NADH/NAD^+^ ratios of 1.0 or higher resembles physiological conditions for S. wolfei cells, although the actual NADH/NAD^+^ ratios during syntrophic growth conditions have not yet been determined.

The partial pressure of hydrogen produced by Hyd1ABC was within the range of the hydrogen partial pressure (25 Pa) produced by suspensions of butyrate-grown S. wolfei cells when conditions allowed only hydrogen production from NADH (27). Butyrate-oxidizing cocultures of S. wolfei and Methanobacterium formicicum maintained a partial pressure of hydrogen of 10.6 Pa (28), while hydrogen concentrations for cocultures of S. wolfei and M. hungatei ranged from 4.6 to 18.0 Pa (Fig. S2). Measurements of partial pressures of hydrogen from methanogenic butyrate-degrading environments, such as lake sediment and sewage sludge, ranged from 3.7 to 26.9 Pa (29). Hydrogen partial pressures from syntrophic, butyrate-oxidizing biogas reactors ranged from 1 to 4.5 Pa (30). Methanogenic syntrophic microcosms established from peat soil implicated members of the Syntrophomonas genus in butyrate oxidation and had hydrogen partial pressures that ranged from 3 to 12.5 Pa (31). Thus, the hydrogen partial pressure that would allow for reoxidation of NADH during syntrophic butyrate metabolism is considerably less than that achieved by confurcating hydrogenases, but it is within the range of the hydrogen partial pressures produced by Hyd1ABC from NADH alone (Table 3). Furthermore, such hydrogen partial pressures are observed in coculture, microcosms, and environments where members of the Syntrophomonas genus would perform syntrophic butyrate oxidation. Thus, the hydrogen partial pressure achieved by Hyd1ABC from NADH would appear to be sufficient to allow syntrophic growth of S. wolfei on butyrate in coculture with a hydrogenotrophic methanogen.

Hyd1ABC was capable of both the reduction of NAD^+^ with hydrogen and the oxidation of NADH to produce hydrogen in the absence of ferredoxin (Fig. 3 and 4; Table 2), indicating the lack of the tight coupling that is reported for bifurcating/confurcating [FeFe]-hydrogenases (11, 14, 15). In addition, Hyd1ABC did not reduce ferredoxin even in the presence of NAD^+^. Thus, if Hyd1ABC is capable of producing hydrogen using electrons from reduced ferredoxin under as-yet-unidentified conditions, the concomitant reduction of NAD^+^ by hydrogen would result in hydrogen being equilibrated with the redox potential of the NADH/NAD^+^ pool, preventing higher hydrogen partial pressures that would normally be achievable by confurcating hydrogenases. It is not surprising that Hyd1 does not interact with ferredoxin. Ferredoxin levels are very low in the proteome of S. wolfei (7), and we did not detect ferredoxin using the traditional approach to purify ferredoxin (N. Losey and M. J. McInerney, unpublished data), e.g., elution from an ion exchange column after high salt treatment and monitoring of the *A*_390_/_280_ ratio (32, 33). Unlike other organisms known to possess confurcating hydrogenases (11, 14–16), S. wolfei does not have a clear mechanism or need to produce reduced ferredoxin in amounts equivalent to the amount of NADH made from butyrate (4, 5). Microorganisms known to possess confurcating [FeFe]-hydrogenases, such as T. maritima (11), M. thermoacetica (15), and R. albus (16), have fermentative metabolisms with ferredoxin-dependent enzymes such as pyruvate:ferredoxin oxidoreductase, which can produce reduced ferredoxin in amounts needed for the coupled hydrogen production from NADH. However, S. wolfei uses fatty acids exclusively as the substrates for growth (1, 21, 34). Beta-oxidation of fatty acids by S. wolfei results in the formation of reduced electron transfer flavoprotein and NADH but not directly in reduced ferredoxin (6).

**FIG 5:**
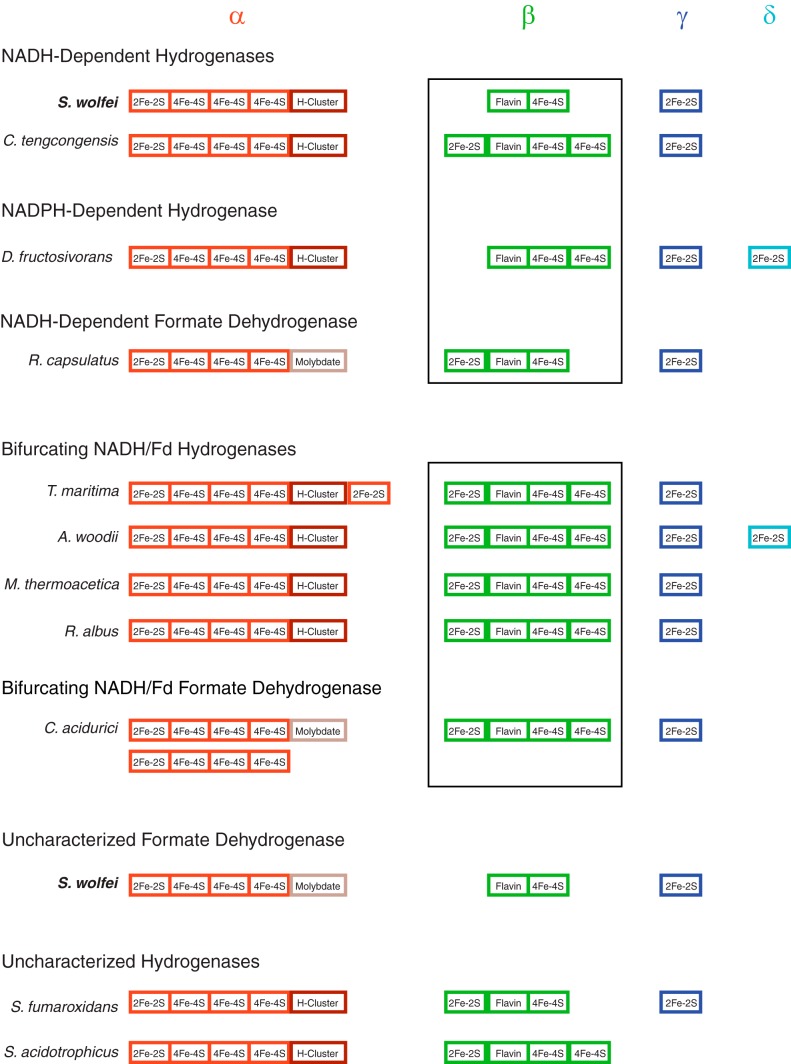
Comparison of predicted conserved domains present in characterized multimeric [FeFe]-hydrogenases and multimeric formate dehydrogenases. The boxes highlight the heterogeneity in the number of conserved domains in HydB (β) subunits of characterized nonbifurcating NADH-dependent [FeFe]-hydrogensases and formate dehydrogenases compared to the similarity of conserved domains of the HydB (β) subunits of characterized bifurcating [FeFe]-hydrogenases and a bifurcating formate dehydrogenase. Heterogeneity in conserved domains is also present in two unstudied [FeFe]-hydrogenases from syntrophic metabolizers S. aciditrophicus and S. fumaroxidans and a formate dehydrogenase from S. wolfei previously found to copurify with Hyd1ABC. The figure is a visual representation of the conserved domains as predicted by NCBI's Conserved Domain Database (40). Greek symbols at the top are the subunit designations. Accession numbers for the sequences of the subunits are given in Table S3. Reported functions of enzymes were obtained from the following references: S. wolfei hydrogenase (this publication), C. tengcongensis (37), D. fructosivorans (12), R. capsulatus (13), T. maritima (11), A. woodii (14), M. thermoacetica (15), R. albus (16), and C. acidurici (36).

The presence of both HydB/HydC subunits has been suggested as being more determining for identifying bifurcating [FeFe]-hydrogenases than the composition of the HydA subunit (22). Compared to the iron-sulfur centers of known bifurcating/confurcating [FeFe]-hydrogenases, the iron-sulfur centers of S. wolfei Hyd1ABC have two differences as predicted by NCBI's Conserved Domain Database (35) that may relate to its inability to use ferredoxin as an electron donor (Fig. 5). The HydB1 subunit lacks an N-terminal [2Fe-2S] cluster and a C-terminal [4Fe-4S] cluster. The difference is reflected in the overall peptide length of S. wolfei HydB1 of 407 amino acid residues compared to the approximately 600 residues for HydB subunits of known bifurcating [FeFe]-hydrogenases. The reportedly nonconfurcating, NADPH-dependent [FeFe]-hydrogenase from Desulfovibrio fructosivorans also lacks an N-terminal [2Fe-2S] cluster in its HydB subunit (Fig. 5) (12). The number of iron-sulfur centers also seems to match with the ability to bifurcate in multimeric molybdopterin formate dehydrogenases. The multimeric, molybdopterin formate dehydrogenase from Clostridium acidurici has been shown to catalyze electron bifurcation and has the same iron-sulfur centers in its HydB and HydC equivalent subunits that known bifurcating [FeFe]-hydrogenases have while a nonbifurcating, NADH-dependent formate dehydrogenase from Rhodobacter capsulatus lacks a C-terminal [4Fe-4S] cluster in its HydB subunit (13, 36). The NADH-dependent [FeFe]-hydrogenase from Caldanaerobacter (formerly Thermoanaerobacter) tengcongensis may be an example of an enzyme whose number of iron-sulfur centers is equivalent to those of bifurcating [FeFe]-hydrogenases but with the ability to reduce NAD^+^ and oxidize NADH without ferredoxin (37). However, the C. tengcongensis [FeFe]-hydrogenase was not tested under bifurcating/confurcating conditions by supplying ferredoxin in the presence of NAD^+^ or NADH. While the number of examples of known bifurcating/confurcating and nonbifurcating [FeFe]-hydrogenases and formate dehydrogenases is small, it appears that the type and number of conserved iron-sulfur domains in the HydB/HydC subunits may be important for electron bifurcation activity. Electron bifurcation involves two independent paths of electron flow, either away from the bifurcation site for bifurcating enzymes or toward the bifurcation site for confurcating enzymes (38, 39). A change in the presence of an iron-sulfur cluster or its ability to facilitate the directionality of electron flow due to a change in redox potential or its distance from other redox centers may affect the bifurcation reaction. It is also possible that the loss of the ability to bifurcate was independent of the change in the number of iron-sulfur clusters and such centers were eliminated over time as they no longer served a critical function.

Multimeric [FeFe]-hydrogenases with the same number of iron-sulfur centers as S. wolfei Hyd1ABC are present in the genomes of other members of the family Syntrophomonadaceae (see Table S3 in the supplemental material). This suggests that Syntrophomonas zehnderi, Syntrophothermus lipocalidus, and Thermosyntropha lipolytica, which perform syntrophic oxidation of fatty acids in coculture with hydrogenotrophic methanogens (40–42), may also utilize nonconfurcating, NADH-dependent hydrogen production. Future studies of additional multimeric [FeFe]-hydrogenases may allow the determination of the relationship, if any, between certain iron-sulfur centers in the HydB and HydC subunits and the ability to perform electron bifurcation.

The [FeFe]-hydrogenase in Syntrophobacter fumaroxidans lacks a C-terminal [4Fe-4S] domain in the HydB subunit, which differs from reported bifurcating [FeFe]-hydrogenases (Fig. 5). However, the metabolism of propionate by the methylmalonyl-CoA pathway by S. fumaroxidans leads to pyruvate formation and the ability to produce reduced ferredoxin, which is required as the low potential donor for electron bifurcation (43). The [FeFe]-hydrogenase in Syntrophus aciditrophicus lacks the HydC subunit, which contains a [2Fe-2S] domain (Fig. 5). S. aciditrophicus may also have the ability to generate reduced ferredoxin if the acyl-CoA dehydrogenases involved in cyclohex-1-ene-1-carboxylate and cyclohexane-1-carboxylate formation can catalyze electron bifurcation (44). The syntrophic metabolism of ethanol and lactate are believed to generate reduced ferredoxin (3, 10, 45). The partial pressures observed during syntrophic ethanol oxidation by cocultures of Pelobacter species with M. hungatei exceeded 1,000 Pa (46), which would not be thermodynamically favorable if NADH was the only electron donor for hydrogen production. Given the higher hydrogen partial pressures and presence of reduced ferredoxin-generating systems, it seems likely that some syntrophic oxidizers may utilize ferredoxin, possibly in a confurcating mechanism, for hydrogen production.

The S. wolfei Hyd1ABC was previously found to copurify with a multimeric formate dehydrogenase encoded by SWOL_RS03955, SWOL_RS03960, SWOL_RS03965, and SWOL_RS03970 (47). The copurification of Hyd1ABC with an apparent formate dehydrogenase suggested a possible formate dehydrogenase-hydrogenase complex, although an alternative would be that Hyd1 and the multimeric formate dehydrogenase are separate complexes that copurify, perhaps due to the similar nature of the HydB/HydC subunits (Fig. 5). Either as separate complexes or as a single larger complex, Hyd1 and the multimeric formate dehydrogenase could function to convert NADH to hydrogen and formate, respectively, which would mean that both metabolites are linked to the NADH/NAD^+^ pool.

The identification of Hyd1ABC as a NADH-dependent hydrogenase suggests that NADH-dependent hydrogen production occurs during syntrophic growth on butyrate by S. wolfei without the need for reduced ferredoxin. Ferredoxin-independent hydrogen production from NADH would allow S. wolfei to avoid the energetically costly reaction to produce reduced ferredoxin from NADH. Based on the hydrogen partial pressures generated by Hyd1ABC (Table 3), continual NADH-dependent hydrogen production would require the presence of a hydrogen-consuming organism to maintain a low hydrogen partial pressure. NADH-dependent hydrogen production could explain in part the obligate requirement S. wolfei has for a hydrogen-consuming organism such as M. hungatei during growth on energetically poor substrates such as butyrate.

## MATERIALS AND METHODS

### Plasmids and strains.

Genes encoding a putative multimeric [FeFe]-hydrogenase were amplified by PCR from S. wolfei genomic DNA with Phusion High-Fidelity DNA polymerase (Thermo Fisher Scientific, Waltham, MA) using the primers listed in Table 4. An amplicon with genes encoding HydA, HydB, and HydC (SWOL_RS05165, SWOL_RS05170, SWOL_RS05175) with the addition of BamHI and AscI restriction sites was inserted into the first multiple cloning site (MCS) of pETDuet-1 (Novagen, Merck KGaA, Darmstadt, Germany) (see Fig. S3 in the supplemental material) with the addition of an N-terminal (His)_6_ residue on HydC to produce pETDuet-1 SwHydABC.

**TABLE 4:** List of primers used for construction of pETDuet-1 SwHydABC, pCDFDuet-1 SwHydEFG, and pCDFDuet-1 SwFd

Primers (F, R)^*a*^	Function
CGCGGATCCGATGATGGATTATAAGGAGATAATTGCCCAG, TTGGCGCGCCCTATAAGAATTTTTTATTCTTGGCATGG	Amplify SWOL_RS05165, SWOL_RS05170, SWOL_RS05175, addition of restriction cut sites for BamHI/AscI
TAACCATATGCAGGATACTCCCAAAGCTA, TAACCTCGAGTTAAAGAATTGCTCGGACCCTG	Amplify SWOL_RS01625, addition of restriction cut sites for NdeI/XhoI
TTCACCATGGAAGCGATATCAGTTAACC, ACATGGATCCTTAAACCTGCTTCAAAGG	Amplify SWOL_RS05180, SWOL_RS05185, SWOL_RS05190, addition of restriction cut sites for NcoI/BamHI
CTTAGGATCCGATGTCTTACATCATC, AGTGATAAGCTTTTAGTCGTCCG	Amplify SWOL_RS010890, addition of restriction cut sites for BamHI/HindIII

a Underlined regions indicate restriction enzyme cut sites. F, forward; R, reverse.

A second plasmid was created to express genes encoding [FeFe]-hydrogenase maturation proteins. The Hyd maturases HydE (SWOL_RS05180) and HydG (SWOL_RS05190), as well as a 348-bp open reading frame with no identifiable conserved domains (SWOL_RS05185), were amplified from the S. wolfei genomic template with the addition of NcoI and BamHI restriction sites and were inserted into the MCS of pCDFDuet-1 (Novagen, Merck KGaA, Darmstadt, Germany) (Fig. S1). In addition, the gene encoding the Hyd maturase HydF (SWOL_RS01625) was amplified with restriction sites for NdeI and XhoI and inserted into the second MCS of pCDFDuet-1 to generate pCDFDuet-1 SwHydEFG.

In addition, a gene encoding a putative ferredoxin, SWOL_RS010890, was amplified by PCR with the addition of BamHI and HindIII restriction sites and inserted into the first MCS of pCDFDuet-1 with the addition of an N-terminal (His)_6_ residue to generate pCDFDuet-1 SwFd. SWOL_RS010890 is predicted to encode a 6-kDa peptide containing a conserved domain for two [4Fe-4S] centers. The SWOL_RS010890 gene product shares 57% amino acid sequence identity with the ferredoxin from Clostridium pasteurianum (GenBank accession number M11214).

Due to changes in the locus tags for S. wolfei genes, a table listing the newly assigned locus tags for S. wolfei used in this publication, older S. wolfei locus tags, and GenBank protein accession numbers is included as Table S4. S. wolfei gene sequences included in this article are available from the S. wolfei genome (GenBank accession number NC_008346), which was published previously (4).

### Expression conditions.

E. coli BL21(DE3) (Thermo Fisher Scientific, Waltham, MA) cells for expression of the genes for His-tagged hydrogenase and His-tagged ferredoxin were grown in LB medium with 50 mM potassium phosphate (pH 7.5) and 10 g · liter^−1^ glucose with appropriate antibiotics at 37°C. Cultures were grown aerobically with shaking at 200 rpm to an optical density (OD)_600_ of 0.4 to 0.6 before induction by the addition of 0.5 mM IPTG (isopropyl-β-d-thiogalactopyranoside), followed by the addition of 2 mM ferric ammonium citrate, 10 mM sodium fumarate, and 2 mM cysteine. After induction, the cultures were sealed with rubber stoppers and sparged with nitrogen gas for 6 h until harvesting. Cells were harvested anaerobically by centrifugation at 6,000 × *g* for 20 min at 4°C, washed by resuspension in 50 mM potassium phosphate (pH 7.5) and then pelleted again by centrifuging at 6,000 × *g* for 10 min at 4°C. The final cell pellets were frozen in liquid N_2_ until further use.

### Purification of His-tagged NADH-dependent hydrogenase.

All manipulations, including cell harvesting and protein purification, were performed inside a Coy chamber with an atmosphere of 95% nitrogen and 1 to 5% hydrogen. All purification buffers were prepared anoxically and contained 2 mM dithioerythritol (DTE) and 5 μM both flavin adenine dinucleotide (FAD) and FMN. Cell pellets (10.8 g) were thawed and suspended in 20 ml of 50 mM Tris-HCl (pH 7.5) with 0.5 M NaCl and 30 mM imidazole, and the cells broken by passage through a French pressure cell operating at an internal pressure of 140 megapascals (MPa). Cell debris was removed by centrifugation at 13,000 × *g* for 20 min at 4°C and passage through a 0.22-μm filter. Resulting cell lysate was applied to a HisTrap HP 5-ml column (GE Healthcare Life Sciences, Pittsburgh, PA) equilibrated with the buffer used for cell breakage, followed by a wash with 50 mM imidazole in the breakage buffer, and His-tagged, NADH-dependent hydrogenase was eluted with 250 mM imidazole in the breakage buffer. Eluted fractions were then concentrated and desalted using an Amicon Ultra 0.5-ml centrifugal filter (EMD Millipore) with a 100-kDa nominal molecular mass limit filter. The desalted fractions were applied to a UNO-Q1 (Bio-Rad, Hercules, CA) column equilibrated with 50 mM Tris-HCl (pH 7.5) and eluted with a gradient of 0 to 0.6 M NaCl over a 17-min period at a flow rate of 2 ml/min. The purified protein was then aliquoted into several vials and flash frozen in liquid nitrogen until used for further analyses.

His-tagged S. wolfei ferredoxin was prepared as described for the His-tagged NADH-dependent hydrogenase, but the purification procedure ended after the HisTrap HP chromatography step.

### Enzymatic assays.

All assays were performed at 37°C unless noted otherwise. Enzyme activity measurements were determined in triplicate with various concentrations of protein to ensure activity was proportional to the amount of protein added. Hydrogenase-oxidizing activity was measured in rubber stopper-sealed, 1.4-ml quartz cuvettes (Nova Biotech, El Cajon, CA) with 600 μl of reaction mix and with hydrogen at a pressure of 1.2 · 10^5^ Pa. The reaction mixture for hydrogen-oxidizing assays consisted of 50 mM Tris-HCl (pH 7.5) or potassium phosphate (pH 7.5), 2 mM DTE, 5 μM FAD, and 5 μM FMN. NAD(P)^+^ reduction was tested with 1 mM NAD(P)^+^ with and without 10 μM C. pasteurianum ferredoxin. Methyl viologen reduction assays were performed using a concentration of 10 mM methyl viologen. Reactions were initiated by either the addition of enzyme or by the addition of 1.2 · 10^5^ Pa of hydrogen to a 100% nitrogen headspace. The kinetic constants for NAD^+^ reduction by hydrogen were determined with various NAD^+^ concentrations (0 to 2.0 mM). Spectrophotometric measurements for the reduction of NAD^+^ and NADP^+^ were performed at 340 nm (ε = 6.2 mM^−1^ cm^−1^). Clostridial ferredoxin reduction was measured at 430 nm (ε_Δox-red_ ≈ 13.1 mM^−1^ cm^−1^) and methyl viologen reduction at 600 nm (ε = 10.0 mM^−1^ cm^−1^).

Hydrogen-producing activities were measured in serum bottles (6.5 ml) sealed with butyl rubber stoppers with a 0.5-ml reaction mixture and shaking at 200 rpm. The reaction mix consisted of 100 mM potassium phosphate at pH 7.5, unless otherwise indicated, as well as 2 mM DTE, 5 μM FAD, and 5 μM FMN. Reactions for hydrogen production were initiated by either the addition of enzyme or the addition of NADH. The headspace was sampled (0.4 ml) every 3.5 min, and percent hydrogen in the gas phase was determined by a gas chromatograph equipped with a reductive gas analyzer. The concentration of NADH and NADPH was 1 mM. A reduced ferredoxin-generating system was used, consisting of 10 mM pyruvate, 1 mM CoA, 0.1 mM thiamine pyrophosphate, 10 μM clostridial or S. wolfei ferredoxin, and 0.1 U of clostridial pyruvate:ferredoxin oxidoreductase.

Assays were set up to determine the final hydrogen concentrations obtained at different NADH/NAD^+^ ratios and pH values. The conditions tested included a NADH/NAD^+^ ratio of either 1.0 or 1.5 with a total pyridine nucleotide pool size of 5 mM with a buffer of 100 mM potassium phosphate at pH 6.5 or 7.0. The ratios of NADH to NAD^+^ used in the above-described experiments were determined from the measured hydrogen partial pressures of growing S. wolfei cultures. Final hydrogen concentrations were determined in duplicate from sealed serum bottles after the reaction mixture was incubated for 24 h at room temperature.

It was previously reported that the presence of trace amounts of viologen dyes in cuvettes and stoppers can decouple bifurcating reactions (48). Hydrogen production assays involving NADH were performed using serum bottles and rubber stoppers that were not previously exposed to viologen dyes. Hydrogen-oxidizing assays were performed with stoppers that were not previously exposed to viologen dyes and with extensively washed cuvettes. Methyl viologen reduction assays were done after all other assays were completed.

### Analytical techniques.

Protein concentration was determined by using the Bradford protein assay (Thermo Fisher Scientific, Waltham, MA) with bovine serum albumin as the standard. SDS-PAGE and native PAGE analyses were performed using precast Novex 8 to 16% Tris-glycine (Life Technologies Co., Carlsbad, CA) according to the manufacturer's instructions. Gels were stained using Coomassie brilliant blue G-250 (Thermo Fisher Scientific, Waltham, MA). Bands from the native gel were excised for peptide identification. The protein in the excised band was digested with trypsin and subjected to high-performance liquid chromatography-tandem mass spectrometry (HPLC-MS/MS) performed by the Laboratory for Molecular Biology and Cytometry Research at OUHSC (Oklahoma City, OK). Resulting peptide fragments were identified by Mascot search and compared to the NCBI nonredundant (nr) database.

Molecular mass of recombinant Hyd1ABC was determined by size exclusion chromatography. Size exclusion chromatography was performed using a Superdex 200 10/300 GL (GE Healthcare Life Sciences) calibrated with gel filtration standards (Bio-Rad Laboratories, Hercules, CA) using a buffer of 50 mM potassium phosphate (pH 7.5) with 0.5 M NaCl at a flow rate of 0.45 ml/min.

Iron content of the purified recombinant Hyd1ABC was determined using a ferrozine assay (49). Identification and quantification of bound flavin were performed by HPLC. The purified recombinant Hyd1ABC was washed 20-fold by ultrafiltration using flavin-free 50 mM Tris-HCl (pH 7.5) and boiled for 10 min, and denatured protein was removed by centrifugation at 13,000 × *g* for 10 min. The supernatant was then analyzed using a Kromasil 100-10-C_18_ column (250 by 4.6 mm) using a buffer of 25% methanol in 50 mM ammonium formate similar to a previous HPLC technique (50) with a UV detector set to 275 nm. Retention times and peak areas were compared to FAD and FMN standards.

Hydrogen concentration was determined by a gas chromatograph (30, 51) equipped with a reductive gas detector Peak Performer RCP-910 (Peak Laboratories, Mountain View, CA). Hydrogen concentrations were determined by comparing peak areas to standards containing hydrogen concentrations of 0.01 to 1%.

### Biochemicals and enzymes.

NADH, NAD^+^, FMN, FAD, pyruvate, thiamine pyrophosphate, methyl viologen, and coenzyme A were purchased from Sigma-Aldrich (St. Louis, MO). Clostridial ferredoxin (33) and pyruvate ferredoxin oxidoreductase (52) were purified from Clostridium pasteurianum (strain DSM 525).

### Culture growth conditions.

Pure cultures of S. wolfei (strain DSM 2245B) and cocultures with M. hungatei strain JF-1 (strain ATCC 27890) were grown anaerobically with resazurin as the redox indicator and cysteine sulfide (0.05%) as the reducing agent. The culture medium consisted of 10 ml · liter^−1^ each of mineral, trace metal, and vitamin solutions (53) prepared anaerobically under a N_2_/CO_2_ (80:20) atmosphere with 3.5 g · liter^−1^ NaHCO_3_ as the buffering agent and 20 mM butyrate or crotonate as the substrate. Cultures were established in triplicate with 160-ml culture medium and 90-ml headspace volumes and incubated at 37°C with shaking at 80 rpm. Growth was monitored at OD_600_ with measurements for fatty acid, hydrogen, and methane concentrations made at 4-day intervals. Determinations of butyrate, crotonate, and methane concentrations were performed by HPLC and gas chromatography-flame ionization detector (GC-FID) as described previously (9) while hydrogen concentrations were determined as described for hydrogen production assays.

## Supplementary Material

Supplemental material
