# Supplementary material for: Syntrophomonas wolfei Uses an NADH-Dependent, Ferredoxin-Independent [FeFe]-Hydrogenase To Reoxidize NADH
Source: Appl Environ Microbiol. 2017 Sep 29;83(20):e01335-17. doi: 10.1128/AEM.01335-17 (PMC5626996; doi:10.1128/AEM.01335-17)
Supplement: Supplemental material [file supp_83_20_e01335-17__index.html]

Supplemental material 

# Syntrophomonas wolfei Uses an NADH-Dependent, Ferredoxin-Independent [FeFe]-Hydrogenase To Reoxidize NADH

## Supplemental material

- Supplemental file 1 -

  Peptide matches obtained from the excised band from native PAGE of the purified, expressed Hyd1ABC (Table S1), comparison of properties of multimeric [FeFe]-hydrogenases (Table S2), GenBank protein accession numbers (Table S3), updated locus tag designations of *S. wolfei* genes (Table S4), kinetics of NAD reduction with hydrogen by Hyd1ABC (Fig. S1), hydrogen partial pressures (Fig. S2), and construction of plasmids (Fig. S3).

  PDF, 462K
