# Supplementary material for: Syntrophomonas wolfei Uses an NADH-Dependent, Ferredoxin-Independent [FeFe]-Hydrogenase To Reoxidize NADH
Source: Appl Environ Microbiol. 2017 Sep 29;83(20):e01335-17. doi: 10.1128/AEM.01335-17 (PMC5626996; doi:10.1128/AEM.01335-17)
Supplement: Supplemental material [file AEM.01335-17_zam999118097s1.pdf]

Table S1. Peptide matches obtained from the excised band from native PAGE of the purified, expressed Hyd1ABC.

Old NCBI Locus Tag	New NCBI Gene Locus	Protein Accession Number	Annotation	Matched peptides	Protein sequence coverage
Swol_1017	SWOL_RS05165	WP_011640436	Hyd1A, iron hydrogenase, small subunit	56	70.6%
Swol_1018	SWOL_RS05170	WP_011640437	Hyd1B, NADH dehydrogense subunit	26	71.4%
Swol_1019	SWOL_RS05175	WP_011640438	Hyd1C, iron-hydrogenase, gamma subunit	5	53.4%
Swol_2436	SWOL_RS12620	WP_011641809	Fe-hydrogenase, large subunit HymC	5	5.7%
Swol_1828	SWOL_RS09445	WP_011641221	NADH dehydrogenase (Quinone)	2	4.2%

Table S2. Comparison of properties of multimeric [FeFe]-hydrogenases

Source organism	Methyl viologen reducing activity (U•mg^{-1}) [a]	NAD^+ reducing activity (U•mg^{-1}) [a]	NADH oxidation activity (U•mg^{-1}) [a]	K_M for NAD^+ (μM)	Molecular weight (kDa)	Assay temperature (°C)	Ref. No.
S. wolfei	571	94.5	6.6	520	124.5	37	This publication
T. maritima	NR	NR [b]	~1.5 [c]	NR	NR	80	1
A. woodii	711	~3.0 [c]	NR	49	300 [d]	30	2
M. thermoacetica	195	57.5 [c]	4.6 [c]	250	300 [d]	45	3
R. albus	NA	60 [c]	NR	NR	NR	37	4
C. tengcongensis	1671	5	10	90	NR	70	5

[a] One unit of activity (U) equals 2 μmol of electrons transferred per min. See the referenced articles for assay conditions.

[b] NR, not reported.

[c] Ferredoxin present.

[d] Reported as a dimer of either αβγ heterotrimer or αβγδ heterotetramer.

Table S3 Genbank protein accession numbers of subunits of multimeric formate dehydrogenases and [FeFe]-hydrogenases.

Source Organism	Subunit	GenBank Protein Accession Number
Acetobacterium woodii	HydA	AFA49450
Acetobacterium woodii	HydB	AFA49451
Acetobacterium woodii	HydC	AFA49452
Acetobacterium woodii	HydD	AFA49454
Caldanaerobacter tencongensis	HydA	AAM24150
Caldanaerobacter tencongensis	HydB	AAM24149
Caldanaerobacter tencongensis	HydC	AAM24148
Clostridium acidurici	FdhA	AFS79904
Clostridium acidurici	FdhA	AFS79905
Clostridium acidurici	FdhB	AFS79906
Clostridium acidurici	FdhC	AFS79907
Desulfovibrio fructosivorans	HydA	AAA87057
Desulfovibrio fructosivorans	HydB	AAA87056
Desulfovibrio fructosivorans	HydC	AAA87054

Desulfovibrio fructosivorans	HydD	AAA87055
Moorella thermoacetica	HydA	ABC20019
Moorella thermoacetica	HydB	ABC20020
Moorella thermoacetica	HydC	ABC20021
Rhodopsuedomonas capsulatus	FdhA	ADE86759
Rhodopsuedomonas capsulatus	FdhB	ADE86760
Rhodopsuedomonas capsulatus	FdhC	ADE86761
Ruminococcus albus	HydA	ADU23430
Ruminococcus albus	HydB	ADU23431
Ruminococcus albus	HydC	ADU23432
Syntrophobacter fumaroxidans	HydA	ABK16541
Syntrophobacter fumaroxidans	HydB	ABK16542
Syntrophobacter fumaroxidans	HydC	ABK16543
Syntrophomonas wolfei	FdhA	ABI68106/ABI68107
Syntrophomonas wolfei	FdhB	ABI68105
Syntrophomonas wolfei	FdhC	ABI68104

Syntrophomonas wolfei	HydA	ABI68331
Syntrophomonas wolfei	HydB	ABI68332
Syntrophomonas wolfei	HydC	ABI68333
Syntrophomonas zehnderi	HydA	WP_046500146
Syntrophomonas zehnderi	HydB	WP_046500148
Syntrophomonas zehnderi	HydC	WP_046500150
Syntrophothermus lipocalidus	HydA	WP_013174729
Syntrophothermus lipocalidus	HydB	WP_013174328
Syntrophothermus lipocalidus	HydC	WP_013174327
Syntrophus aciditrophicus	HydA	ABC76974
Syntrophus aciditrophicus	HydB	ABC76975
Thermosyntropha lipolytica	HydA	WP_073091114
Thermosyntropha lipolytica	HydB	WP_073091112
Thermosyntropha lipolytica	HydC	WP_073091109
Thermotoga maritima	HydA	AAD36496
Thermotoga maritima	HydB	AAD36495

Thermotoga maritima	HydC	AAD36494

Table S4. Updated locus tag designations of *S. wolfei* genes.

Old NCBI Locus Tag	New NCBI Gene Locus	Protein Accession Number	Genome Location
Swol_1017	SWOL_RS05165	WP_011640436	NC_008346 REGION: 1157124.1158848
Swol_1018	SWOL_RS05170	WP_011640437	NC_008346 REGION: 1158873.1160096
Swol_1019	SWOL_RS05175	WP_011640438	NC_008346 REGION: 1160156.1160602
Swol_1020	SWOL_RS05180	WP_041427405	NC_008346 REGION: 1160971.1162029
Swol_1021	SWOL_RS05185	WP_041427406	NC_008346 REGION: 1161998.1162378
Swol_1022	SWOL_RS05190	WP_011640441	NC_008346 REGION: 1162353.1163816
Swol_0318	SWOL_RS01625	WP_011639769	NC_008346 REGION: 384562.385770
Swol_2105	SWOL_RS10890	WP_011641488	NC_008346 REGION: 2424114.2424290
Swol_1925	SWOL_RS09950	WP_011641315	NC_008346 REGION: 2197847.2199010
Swol_2436	SWOL_RS12620	WP_011641809	NC_008346 REGION: 2765177.2766868

Fig. S1. Kinetics of NAD^+ reduction with hydrogen by Hyd1ABC. Reactions were performed at 37°C in 1.6 ml cuvettes with 800 µl of 50 mM potassium phosphate buffer (pH 7.5), with 5 µm FAD, 5 µm FMN and 86 µg of Hyd1ABC. The data were fit to the reaction curve using non-linear regression with a K_m of 0.52 mM NAD^+ and a V_{max} of 196 µmole•min^{-1}•mg^{-1}of protein. A k_{cat} of 406.7 s^{-1} was determined using a single catalytic site per a molecular mass of 124.5 kDa.

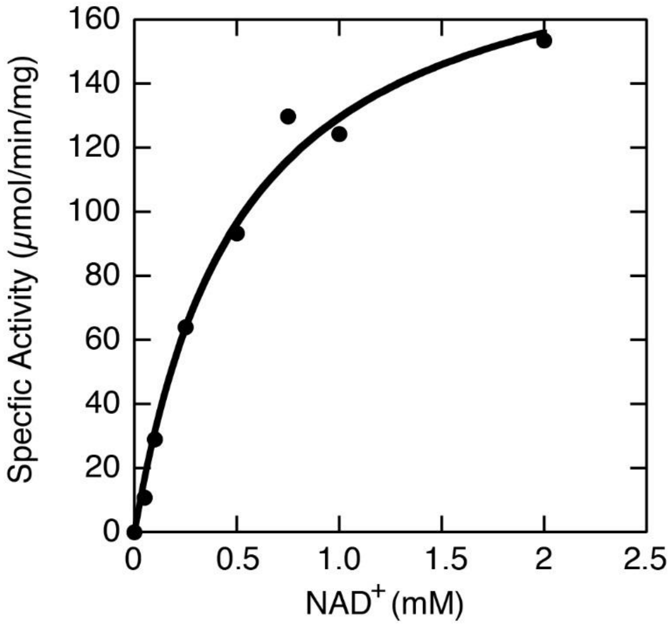

Fig. S2. Hydrogen partial pressures during growth of *S. wolfei* in pure culture and in coculture with *M. hungatei*. (A and B) *S. wolfei* grown in pure culture on crotonate; (C and D) *S. wolfei* grown in coculture with *M. hungatei* on crotonate; (E and F) *S. wolfei* grown in coculture with *M. hungatei* on butyrate. A, C, E, growth (diamonds) and hydrogen (Pa) (triangles); (B, D, F) substrate concentration (crotonate in B and D and butyrate in F) (circles) and methane concentration (squares).

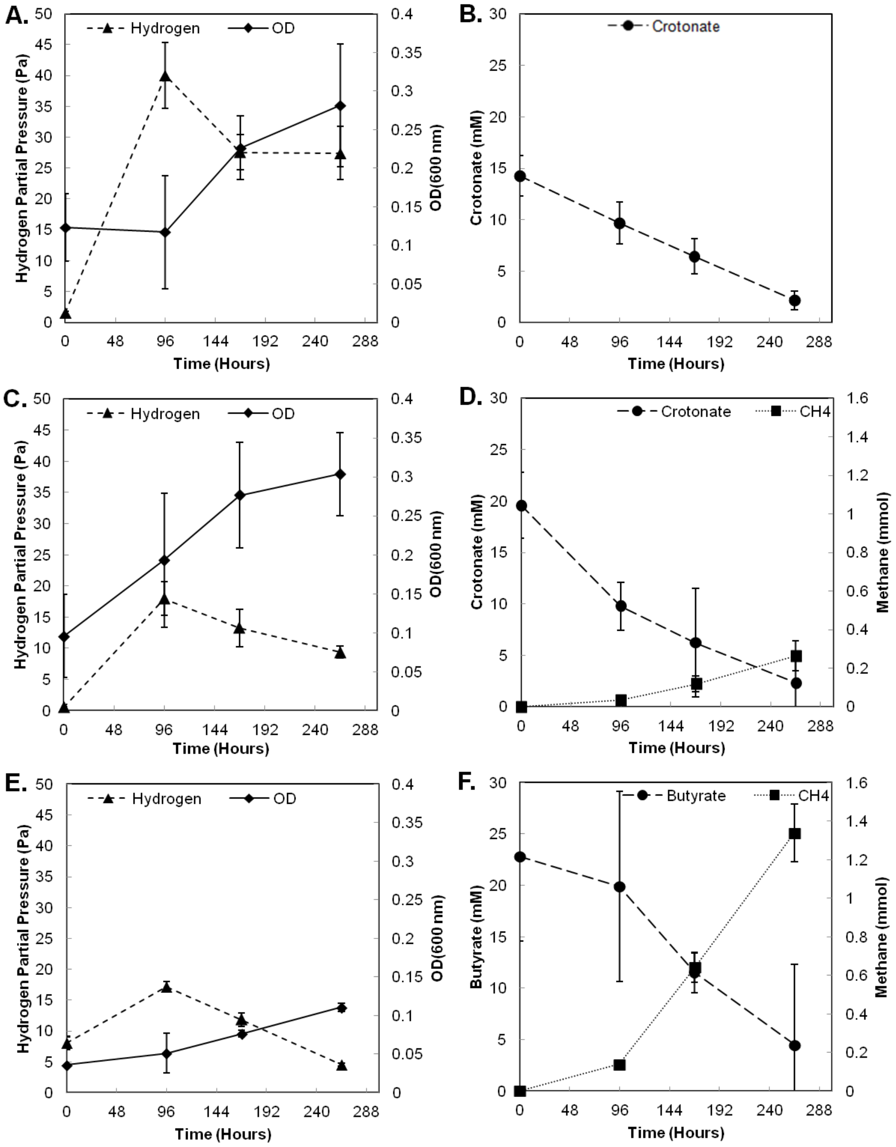

Fig. S3. Construction of plasmids for expression of Hyd1ABC and [FeFe]-hydrogenase maturation proteins.

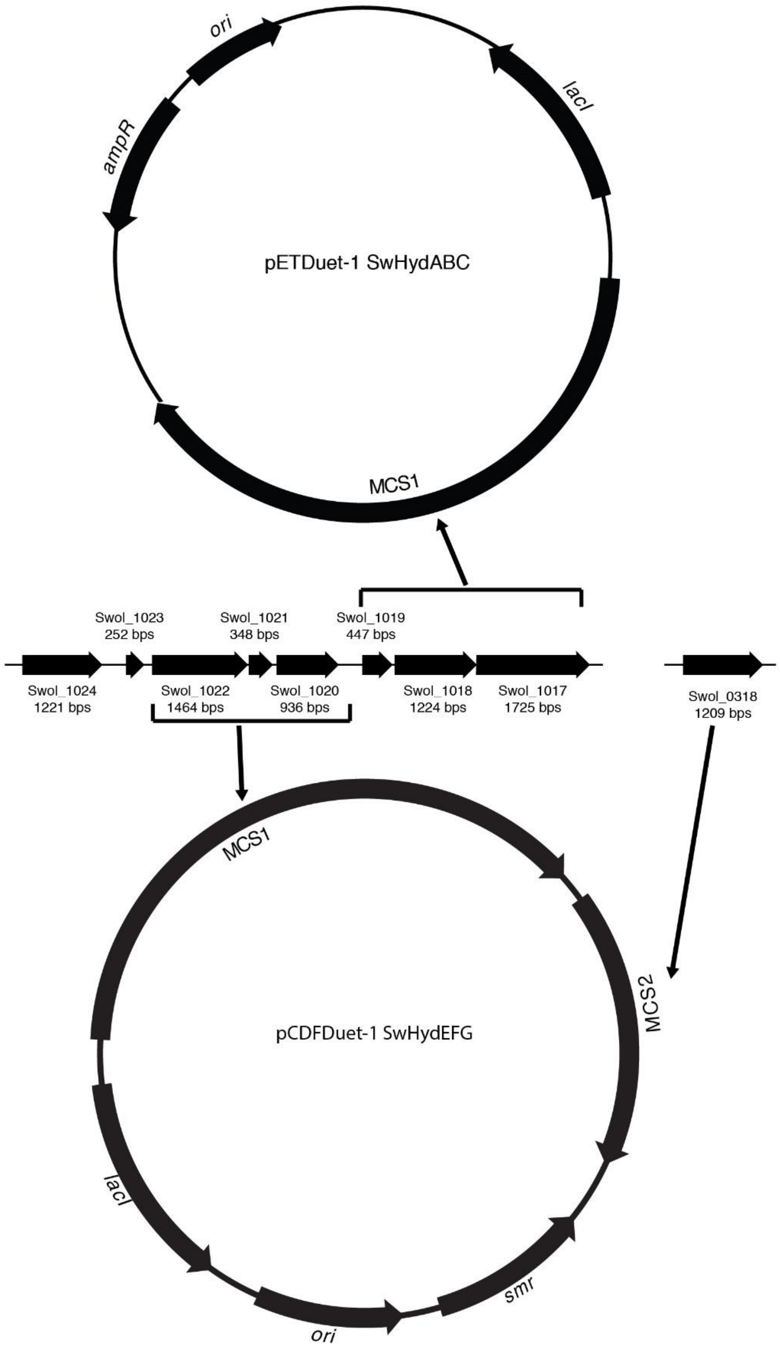

References

1. **Schut GJ, Adams MW.** 2009. The iron-hydrogenase of *Thermotoga maritima* utilizes ferredoxin and NADH synergistically: a new perspective on anaerobic hydrogen production. J Bacteriol **191:**4451-4457.
2. **Schuchmann K, Müller V.** 2012. A bacterial electron-bifurcating hydrogenase. J Biol Chem **287:**31165-31171.
3. **Wang S, Huang H, Kahnt J, Thauer RK.** 2013. A reversible electron-bifurcating ferredoxin- and NAD-dependent [FeFe]-hydrogenase (HydABC) in *Moorella thermoacetica*. J Bacteriol **195:**1267-1275.
4. **Zheng Y, Kahnt J, Kwon IH, Mackie RI, Thauer RK.** 2014. Hydrogen formation and its regulation in *Ruminococcus albus*: involvement of an electron-bifurcating [FeFe]-hydrogenase, of a non-electron-bifurcating [FeFe]-hydrogenase, and of a putative hydrogen-sensing [FeFe]-hydrogenase. J Bacteriol **196:**3840-3852.
5. **Soboh B, Linder D, Hedderich R.** 2004. A multisubunit membrane-bound [NiFe] hydrogenase and an NADH-dependent Fe-only hydrogenase in the fermenting bacterium *Thermoanaerobacter tengcongensis*. Microbiology **150:**2451-2463.
